# Microsatellite Instability in Mouse Models of Colorectal Cancer

**DOI:** 10.1155/2018/6152928

**Published:** 2018-03-01

**Authors:** Nicola Currey, Joseph J. Daniel, Dessislava N. Mladenova, Jane E. Dahlstrom, Maija R. J. Kohonen-Corish

**Affiliations:** ^1^The Kinghorn Cancer Centre, Garvan Institute of Medical Research, Sydney, NSW, Australia; ^2^ACT Pathology, The Canberra Hospital and Australian National University Medical School, Canberra, ACT, Australia; ^3^St. Vincent's Clinical School, UNSW Medicine, UNSW Sydney, Sydney, NSW, Australia; ^4^School of Medicine, Western Sydney University, Campbelltown, NSW, Australia

## Abstract

Microsatellite instability (MSI) is caused by DNA mismatch repair deficiency and is an important prognostic and predictive biomarker in colorectal cancer but relatively few studies have exploited mouse models in the study of its clinical utility. Furthermore, most previous studies have looked at MSI in the small intestine rather than the colon of mismatch repair deficient* Msh2*-knockout (KO) mice. Here we compared* Msh2*-KO,* p53*-KO, and wild type (WT) mice that were treated with the carcinogen azoxymethane (AOM) and the nonsteroidal anti-inflammatory drug sulindac or received no treatment. The induced tumors and normal tissue specimens from the colon were analysed with a panel of five mononucleotide repeat markers. MSI was detected throughout the normal colon in untreated* Msh2*-KO mice and this involved contraction of the repeat sequences compared to WT. The markers with longer mononucleotide repeats (37–59) were the most sensitive for MSI while the markers with shorter repeats (24) showed only minor change. AOM exposure caused further contraction of the* Bat37* and* Bat59 *repeats in the distal colon of* Msh2*-KO mice which was reversed by sulindac. Thus AOM-induced carcinogenesis is associated with increased instability of mononucleotide repeats in the colon of* Msh2*-KO mice but not in WT or* p53*-KO mice. Chemoprevention of these tumors by sulindac treatment reversed or prevented the increased MSI.

## 1. Introduction

Microsatellite instability (MSI) in cancer occurs as a result of frameshift mutations in tandem repeat sequences known as microsatellites. These replication errors are caused by polymerase slippage and are normally corrected by the DNA mismatch repair (MMR) proteins; however impairment of this pathway through loss of its components prevents this correction and causes the accumulation of MSI throughout the genome in cancer cells, including coding and noncoding sequences [1].

MMR is a highly conserved cellular process that involves several protein complexes [2]. In the eukaryotes, there are two major MutS heterodimers, MSH2/MSH6 (MutS*α*) and MSH2/MSH3 (MutS*β*), which bind to DNA and initiate the repair of the insertion-deletion-loop mismatches. The MutL*α* complex comprises MLH1/PMS2 and has a coordinator role in MMR. Germline mutations in the MMR genes* MLH1*,* MSH2*,* MSH6*, and* PMS2 *cause Lynch syndrome, which has a lifetime colorectal cancer risk of up to 80%, and is characterised by MSI in the tumors [3]. MSI is also found in sporadic colon cancers as a result of methylation silencing of* MLH1*. High-level MSI (MSI-H) is associated with various clinicopathological and molecular features, including proximal location of tumors,* BRAF* mutation, CpG island methylator phenotype (CIMP), and good prognosis [4–6]. MSI-positive colorectal cancers are more responsive to immunotherapy involving immune checkpoint blockade, possibly due to an increased level of mutation-associated neoantigens [7].

In mice, constitutive deficiency of individual MMR proteins also causes cancer development, but the spontaneous tumors develop in the small intestine rather than the colon. Relatively few studies have looked at MSI in the mouse colon or compared MSI in the normal colon of* Msh2*-knockout (KO) and wild type (WT) mice, while there are several reports on the small intestine [8, 9]. Methods to chemically induce tumorigenesis have been developed in order to model colorectal tumors in mice. Administration of the carcinogen azoxymethane (AOM) causes multiple tumors in the distal colon and this is prevented or reduced by sulindac, a nonsteroidal anti-inflammatory drug (NSAID) [10, 11]. In our previous study we showed that both* Msh2*-KO and* p53*-KO mice have an increased susceptibility to AOM-induced colon carcinogenesis and that sulindac is protective in the distal colon but has an adverse effect in the proximal colon [11]. AOM is known to cause at least low-level MSI in the colon tumors of WT mice but there are a few studies on MSI in AOM-induced tumors in MMR or p53 deficient mice [12]. Therefore, this comparative study aimed to examine MSI in the normal colon and distal tumors in WT,* Msh2*-KO, and* p53*-KO mice exposed to AOM and sulindac.

## 2. Materials and Methods

### 2.1. Mouse Breeding and Experimentation

The whole body knockout lines* Msh2*-KO and* p53*-KO were maintained in a Specific Pathogen Free breeding facility, crossing heterozygotes with wild type C57Bl6J strain. The* Msh2*-KO mice were initially generated in the 129/OLA strain [13]. Here we analysed* Msh2*-KO and* p53*-KO mice from our previous study, all on the C57Bl6J background [11], as well as additional* Msh2*-KO mice and their WT siblings, which had a mixed strain background. The mice received three injections of AOM (15 mg/kg at 8, 9, and 10 weeks) and feed containing sulindac for 22 weeks (160–320 ppm) and were all culled at 28 weeks of age. Altogether, we analysed 8–13 AOM-treated WT,* p53*-KO, or* Msh2*-KO mice and 17* Msh2*-KO mice that received both AOM and sulindac. The macroscopic and histopathology analysis of the colon tissue specimens has been previously described in detail. The animal experimentation was approved by the Animal Ethics Committees of the Central Sydney Area Health Service (99/99/MCG/01) and Garvan Institute of Medical Research (02/05; 05/07).

### 2.2. Preparation of Tissue Specimens

Archival tissue samples were selected that would reflect a wide range of treatment conditions, genotypes, and pathological stages. Multiple specimens were analysed for some mice. These included adenocarcinomas and dysplastic tumors from the distal colon and normal tissue from different parts of the colon. Specimens were manually microdissected from formalin-fixed paraffin embedded tissue that had been scored by a specialist Anatomical Pathologist (JED). DNA was isolated using a modified protocol of the Gentra Puregene Tissue Kit (Qiagen).

### 2.3. Analysis of MSI

For MSI analysis, we chose four mononucleotide repeat markers that were identified by Bacher et al. [14] as sensitive indicators of MSI in the mouse,* Bat24*,* Bat37*,* Bat59*, and* Bat64*, where the number indicates the length of the poly-A repeat (24–64). We also included the 24-T repeat marker* uPAR*, which we used previously in the analysis of colitis-associated cancers [15]. PCR amplification was carried out in a multiplex reaction using MyTaq polymerase (Bioline), with primer concentrations ranging from 0.1 to 0.4 *μ*M. The thermal cycling conditions were as follows: initial denaturation at 95°C for 10 minutes; followed by 40 cycles of 95°C for 45 seconds, 55°C for 45 seconds, and 68°C for 1 minute; then a final extension step at 68°C for 10 minutes. PCR fragments were analysed by capillary electrophoresis, ABI3130XL (Life Technologies), and the GeneMapper program (Life Technologies).

### 2.4. Definition of MSI for Individual Markers

Each marker pattern in the test specimen was compared to the appropriate control tissue as indicated in the results. As some further instability can appear as a result of the polymerase slippage during PCR, the whole pattern of major and minor peaks was compared. MSI-positivity was scored when at least a 1 bp contraction of the repeat was observed, (i.e., deletion, shown as a shift of the highest peak), or if new peaks appeared that were not present in the control tissue. A “minor shift” was recorded, when the tallest peak remained the same but there was otherwise a shift in the overall MSI pattern.

### 2.5. Statistical Analysis

Graphpad Prism software (GraphPad Inc.) was used for statistical analyses. Unpaired *t*-test was used to determine the statistical significance of the repeat length variation between genotype and treatment groups.

## 3. Results

### 3.1. MSI in the Normal Colon of the Msh2 Knockout Mouse

**Figure 1:**
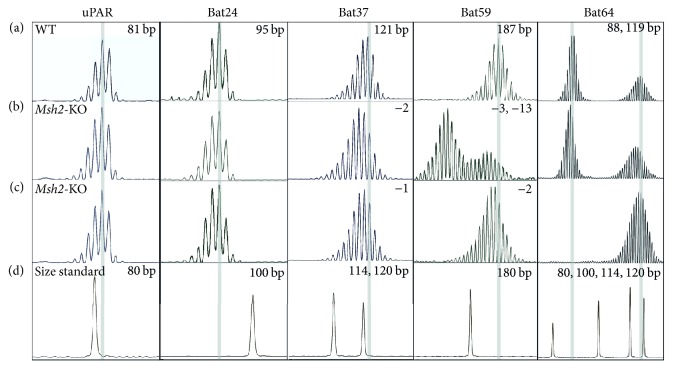
MSI in the normal colon of the* Msh2*-KO mouse. (a) Reference pattern of mononucleotide repeat markers* uPAR*,* Bat24*,* Bat37*,* Bat59*, and* Bat64* in a WT mouse.* Bat64* shows two alleles. The position of the highest peak in the WT mouse is highlighted with the grey bar and the size is indicated in the top right corner; (b, c) contraction of the repeats in two representative* Msh2*-KO mice compared to WT. (d) The position and size of the peaks for the size standard ROX.

**Figure 2:**
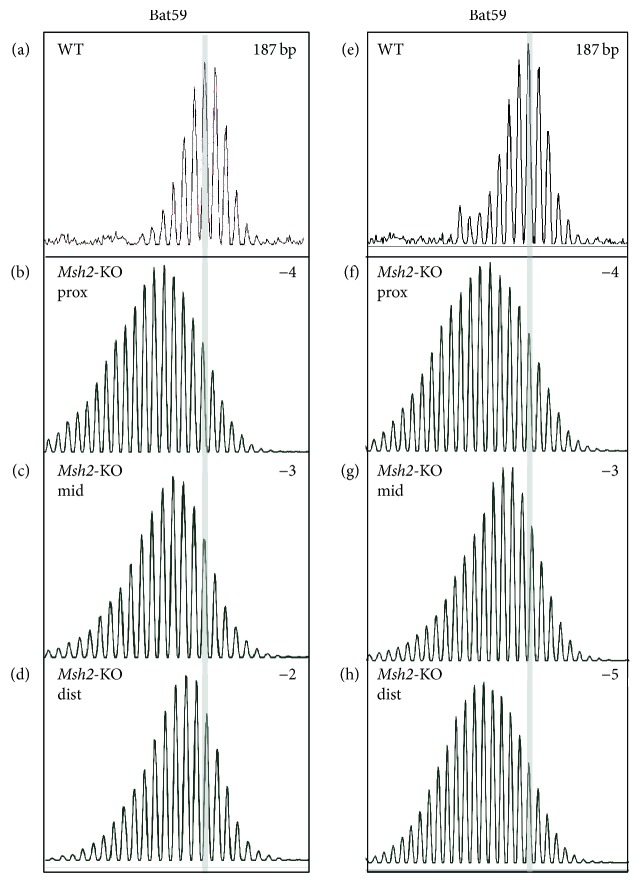
The length of the* Bat59* mononucleotide repeat varies slightly within individual* Msh2*-KO mice. (a, e)* Bat59* in the colon of two WT mice; (b–d); (f–h)* Bat59* in the proximal, mid, and distal colon of two* Msh2*-KO mice. The position of the highest peak in the WT mouse is highlighted with the grey bar; 187 bp refers to the size of the PCR fragment in the WT and the number in the top right corner indicates the number of nucleotides by which the repeat is shortened in each* Msh2*-KO mouse.

We analysed MSI in* Msh2*-KO and* p53*-KO mice that did not receive any specific treatment and compared the pattern of instability to their WT siblings. The* Msh2* or* p53* deficient mice are susceptible to carcinogenesis in various organs but do not usually develop spontaneous colon tumors [13, 16]. MSI was only observed in the* Msh2*-KO mice where a subset of the markers detected consistent instability in the normal colon (Figure 1). This involved contraction of the repeats compared to WT, but there was variation in the length of the deletion for each marker.* Bat37* revealed deletions of 1-2 bp while* Bat59* showed deletions of 2–5 nucleotides in 8 of the 9 mice (Figure 2) and additional new peaks in one mouse (~13 nucleotides shorter). The differences between the WT and* Msh2*-KO mice were statistically significant, *P* < 0.001. The shorter repeats were not as sensitive indicators of MSI as the longer repeats. The* Msh2*-KO specimens commonly showed a minor shift in the overall MSI pattern for the* uPAR* and* Bat24* markers. Therefore, in subsequent analyses, we focused on* Bat37* and* Bat59*. One WT mouse showed two alleles for* Bat59*, but all other mice appeared monomorphic.* Bat64 *was found to be polymorphic in the* Msh2*-KO and* p53*-KO lines (Figure 1). Altogether, we identified four alleles in the group of mice studied here, 80 bp for the 129/OLA strain and 88, 103, or 119 bp for C57Bl6J. This indicates that there is genetic variation for this repeat even within C57Bl6J.

### 3.2. MSI in Mice Exposed to AOM and Sulindac

Injection of the carcinogen AOM causes the development of multiple tumors in the mouse distal colon. Our previous study showed that these tumors feature low- to high-grade dysplasia (LGD/HGD) or adenocarcinoma. The combined frequency of HGD and adenocarcinoma was 35% in WT, 45% in* Msh2*-KO, and 63% in* p53*-KO mice [11].* Msh2*-KO and* p53*-KO groups developed significantly more and larger tumors per mouse than the WT group [11].

**Figure 3:**
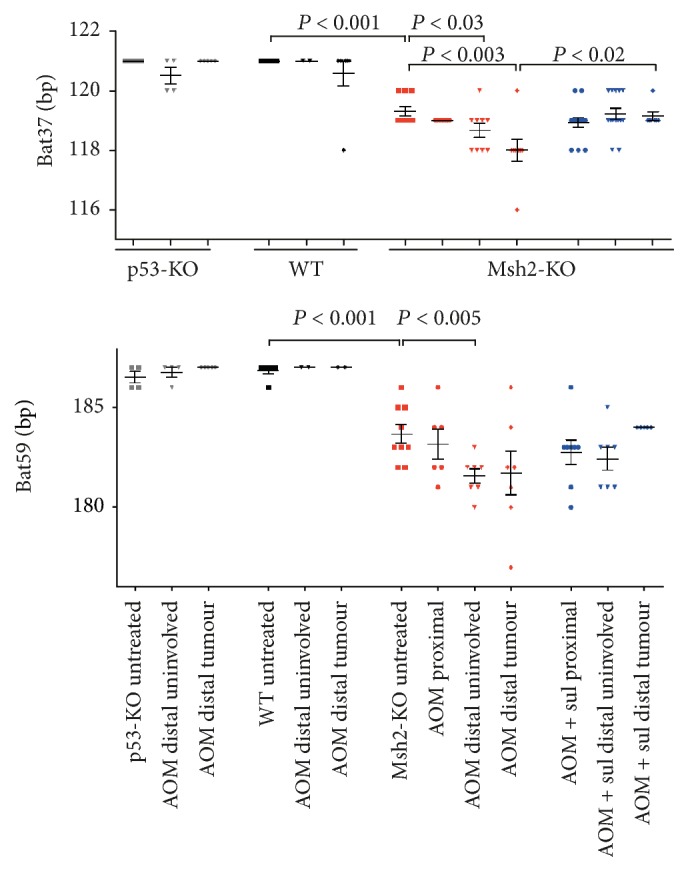
AOM exposure causes a further contraction of the* Bat37* and* Bat59 *repeats in the distal colon of* Msh2*-KO mice which is reversed by sulindac (sul) treatment. The error bars indicate SEM.* p53*-KO (grey), WT (black),* Msh2*-KO, AOM (red), and* Msh2*-KO, AOM + sulindac (blue).

**Figure 4:**
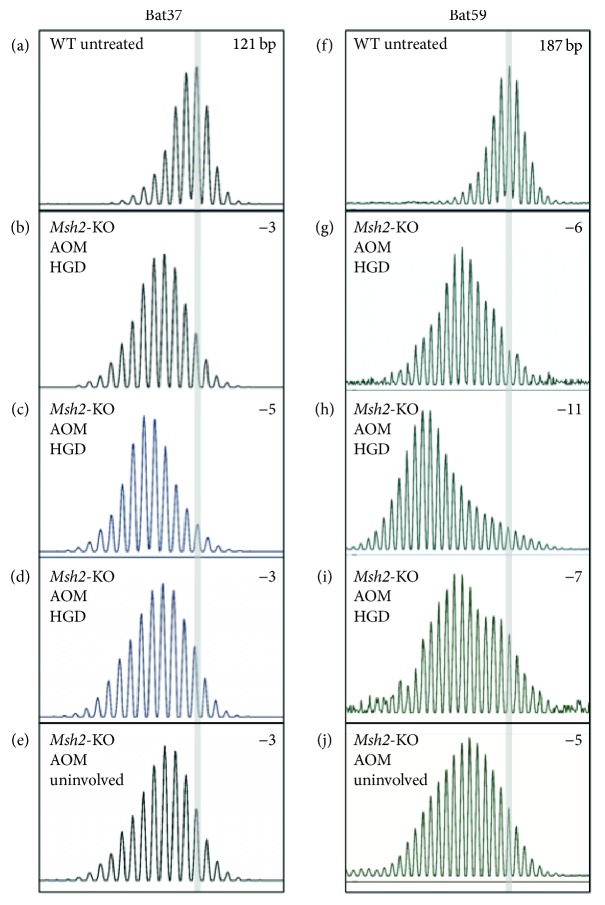
AOM-treated* Msh2-*KO mice display further contraction of the mononucleotide repeats in the distal colon. (a, f)* Bat37* and* Bat59* in the colon of a WT mouse; (b–d); (g–i) AOM-induced tumors from a* Msh2*-KO mouse; (e, j) uninvolved colon tissue from an AOM-treated* Msh2*-KO mouse. The position of the highest peak in the WT mouse is highlighted with the grey bar; 121 and 187 bp refer to the size of the PCR fragment in the WT and the number in the top right corner indicates the number of nucleotides by which the repeat is shortened in the* Msh2*-KO mouse.

Here we first analysed MSI in a subset of the AOM-treated mice that developed either HGD or adenocarcinoma. The* Msh2*-KO mice showed MSI, which appeared to involve additional contraction of the mononucleotide repeats. The length of the deletions was variable, 3–10 bp for* Bat59* and 2–5 bp for* Bat37*, compared to WT (Figures 3 and 4). This additional contraction of the two repeats was detected in both the tumors and uninvolved tissue from the distal colon of AOM-treated mice and was not observed in the proximal colon.

Administration of dietary sulindac to AOM-treated mice can prevent the tumors from developing in the distal colon. In our previous experiment this treatment completely prevented neoplastic distal tumors in WT and lowered the frequency of HGD/adenocarcinoma to 3% in* Msh2*-KO and 7% in* p53*-KO mice [11]. Here we analysed the effect of this double treatment on MSI. Sulindac reversed the additional contraction of* Bat37* and* Bat59* in the distal colon of AOM-treated* Msh2*-KO mice. This was evident in both the few remaining tumors and in the uninvolved surrounding tissue of the distal colon. There was no change in the uninvolved tissue of the proximal colon in mice exposed to both AOM and sulindac compared to AOM alone (Figure 3).

## 4. Discussion

This study used a panel of proven mononucleotide repeat markers to analyse MSI in the mouse colon. MSI was not observed in the chemically induced colon cancers in most WT and* p53*-KO mice. Thus, p53 deficiency promotes AOM-induced colon carcinogenesis [11], but this does not commonly involve instability of mononucleotide repeats. It is possible that these tumors still harbor a defect in the MMR process that repairs dinucleotide or tetranucleotide repeats, which were not analysed here. Low-level MSI of dinucleotide repeats was previously demonstrated in AOM-induced colon tumors in the A/J WT strain [12].

In contrast, MSI was commonly observed in the normal colon of* Msh2*-KO mice that do not develop colon cancer spontaneously. This instability was found to a varying degree with each marker.* Msh2* deficiency clearly causes contraction of the mononucleotide repeats* Bat37* and* Bat59* in the normal mouse colon, but the deletions were not as extensive in the shorter repeats (*uPAR* and* Bat24*). Here we also demonstrate that further contraction of the* Bat37* and* Bat59* repeats occurs in the distal colon of AOM-treated* Msh2*-KO mice. This effect was not seen in the proximal colon, which is consistent with the selectivity of AOM to the distal colon.

We could not find a previous report in the literature analysing MSI in the normal colon of* Msh2*-KO mice. The* Bat24*,* Bat37,* and* Bat59* markers were used by Bacher et al. [14] to show instability in the spontaneous tumors that develop in the small intestine of the* Msh2*-KO mouse. It remains unexplained why these mice do not develop spontaneous tumors in the colon. However, this is likely to require the generation of further cancer driver mutations. The coding regions of genes usually only harbor short repeat sequences, such as the 10-A and 8-G repeats in* TGFBR2* and* BAX* genes, respectively, that are frequently mutated in MSI-H human tumors [17]. Our data show that the two markers with 24-mononucleotide repeats were less affected by* Msh2* deficiency in the colon than the 37–59-mononucleotide repeats. Therefore, it is possible that the coding regions of genes are also less frequently mutated in the colon of these mice.

We also report for the first time that exposure to the NSAID sulindac can reverse the increased instability that was caused by AOM in* Msh2*-KO mice. This was observed in the specimens obtained from the distal colon of AOM-treated mice and was most prominent for the* Bat37* and* Bat59* markers. This may indicate an opposite effect on the repeat length, contraction for AOM and expansion for sulindac exposure* in vivo*. Alternatively sulindac treatment may selectively target cells with MSI. It was previously described that aspirin, another NSAID, can reduce MSI* in vivo* in the intestinal epithelium of mice with* Msh2 *deficiency [18]. Aspirin has also been found effective in reducing the risk of colorectal cancer in carriers of Lynch syndrome mutations [19].* In vitro* studies with both mouse and human MMR deficient cells indicated that this may be due to NSAID-mediated apoptosis that selectively targets cells with MSI, thus having an overall stabilizing effect on the repeats [20, 21]. However, it should be noted that, in our study, AOM-induced distal colon tumors were also prevented by sulindac in WT mice and on the background of* p53* deficiency [11], in the absence of mononucleotide repeat instability. This indicates that there are multiple chemopreventive mechanisms.

In conclusion, instability of mononucleotide repeats is present in the normal colon of* Msh2*-KO mice that do not develop spontaneous colon cancers. AOM exposure caused further contraction of the repeats in* Msh2*-KO mice but this was reversed by administration of dietary sulindac in both the AOM-induced tumors and uninvolved tissue of the distal colon. There was no MSI found in the AOM-induced colon tumors in the* p53*-KO and WT mice.
